# Linking Peripartal Dynamics of Ruminal Microbiota to Dietary Changes and Production Parameters

**DOI:** 10.3389/fmicb.2016.02143

**Published:** 2017-01-12

**Authors:** Hooman Derakhshani, Hein M. Tun, Felipe C. Cardoso, Jan C. Plaizier, Ehsan Khafipour, Juan J. Loor

**Affiliations:** ^1^Department of Animal Science, University of ManitobaWinnipeg, MB, Canada; ^2^Department of Animal Sciences, University of IllinoisUrbana, IL, USA; ^3^Department of Medical Microbiology, University of ManitobaWinnipeg, MB, Canada; ^4^Division of Nutritional Sciences and Illinois Informatics Institute, University of IllinoisUrbana, IL, USA

**Keywords:** nutrition, transition period, rumen microbiota, 16S rRNA gene sequencing, dairy cattle

## Abstract

During the peripartal period, proper acclimatization of rumen microorganisms to variations in nutritional management can facilitate the transition into lactation. This study characterized the temporal shifts in the composition and functional properties of ruminal microbiota during the periparturient period in dairy cows subjected to a typical two-tiered feeding management approach. Ruminal digesta samples from eight multiparous fistulated Holstein cows were collected on days −14, −7, 10, 20, and 28 relative to parturition. High-throughput Illumina sequencing of the V4 region of the bacterial 16S rRNA gene revealed distinct clustering patterns between pre- and postpartal ruminal microbiota. During the prepartal period, when the voluntary dry matter intake was lower, we observed strikingly lower inter-animal variations in the composition of the ruminal microbiota. Genera *Ruminococcus* and *Butyrivibrio*, which are considered major fibrolytic rumen dwellers, were overrepresented in the prepartal rumen ecosystem. In contrast, increased postpartal voluntary DMI was associated with enrichment of bacterial genera mainly consisting of proteolytic, amylolytic, and lactate-producer species (including *Prevotella, Streptococcus*, and *Lactobacillus*). These, together with the postpartal enrichment of energy metabolism pathways, suggested a degree of acclimatization of the ruminal microbiota to harvest energy from the carbohydrate-dense lactation diet. In addition, correlations between ruminal microbiota and parameters such as milk yield and milk composition underscored the metabolic contribution of this microbial community to the cow's performance and production.

## Introduction

Nutritional management of dairy cattle during the peripartal period, i.e. the last 3 weeks before and the first 3 weeks after parturition, can impact the ability of the animal to make a successful transition into lactation by remaining free of health disorders while optimizing nutrient use for production (Loor et al., 2013; Roche et al., 2013). Adaptations to nutritional management occur at the endocrine, metabolic, and molecular levels (Loor et al., 2013). Furthermore, ruminal papillae in non-lactating/pregnant cows require 4–7 weeks to fully adapt to the higher-energy diets required by the animal after parturition. As such, prepartal feeding management could increase the capacity for volatile fatty acid (VFA) absorption postpartum (Mayer, 1986), when the cow's ability to consume dry matter is most compromised. The two-tiered feeding approach that is commonly utilized in the dairy cattle industry for late-pregnant cows was meant to provide time for the ruminal microbiome to “adapt” to the dietary changes occurring after parturition (Kertz et al., 1991), and also to enhance the production of VFA via fermentation of non-structural carbohydrate (e.g., starch) for optimal ruminal papillae development. In theory, this period of adaptation to higher-energy diets encompassing the last 3–4 weeks prepartum should help diminish the incidence of ruminal disorders especially after calving.

Recent advances in molecular microbiology and high throughput sequencing have enhanced our understanding of the complex web of microbial symbionts—microbiota—that inhabit the anaerobic environment of rumen (McCann et al., 2014). Members of ruminal microbiota perform a number of essential protective, structural, and metabolic functions of relevance to animal physiology and performance, including metabolism of dietary nutrients, degradation and fermentation of complex indigestible polysaccharides, and synthesis of vitamins. Metabolic end products of ruminal microbiota, such as short-chain fatty acids (SCFAs), can be absorbed through the rumen wall and help meet the energy requirements of the ruminant (Shabat et al., 2016). In fact, by providing a secondary genome—metagenome—to their mammalian hosts, commensal bacteria can influence the general physiology and performance to a degree that they have been considered as virtual endocrine organs (Evans et al., 2013).

Although the endocrine, metabolic, physiologic, and molecular adaptations occurring in dairy cows around the peripartal period are reasonably well-described (Loor et al., 2013), there are few published studies (Lima et al., 2015; Pitta et al., 2014a; Indugu et al., 2016) that have evaluated the shifts in the ruminal microbiota composition during this period. More importantly, there is a knowledge gap on the changes occurring in the functional properties of the ruminal microbiota during the transition into lactation and their correlation with phenotypic measures of animal performance traits, such as milk production and composition. Thus, the main objectives of this study were to (a) characterize changes in the ruminal microbiota composition during the peripartal period, (b) predict shifts in the functional properties of the ruminal microbiome during this period, and (c) correlate such changes with dry matter intake (DMI), milk production, and milk composition.

## Materials and methods

All animal experimental procedures were performed under protocols approved by the University of Illinois Institutional Animal Care and Use Committee (protocol #12094).

### Animal experiment and sampling

Eight ruminally-cannulated Holstein cows in their third or greater lactation were used. Cows were managed according to the University of Illinois Dairy Research Farm standard operating procedures, and fed during the dry period using the two-stage approach with a high wheat straw, lower-energy diet from dry-off through d −21 from parturition followed by a low wheat straw, higher-energy (by increasing corn grain) diet from d −21 until parturition. Cows were then fed a common lactation diet until d 30 postpartum (Table 1). Diets were fed as a total mixed ration (TMR) once daily (0600 h) using an individual Calan gate feeding system (American Calan, Northwood, NH, USA) during the dry period or in tie-stalls during lactation. Collection of body weight (BW), body condition score (BCS), milk production data and sampling for milk composition analysis was as described previously (Graugnard et al., 2013). Briefly, cow BW were obtained weekly, and BCS assigned weekly throughout the study. Cows were milked three times daily after parturition. Milk was sampled thrice weekly at each milking, and samples were pooled within day of collection and across week to generate a sample for analysis of chemical composition.

**Table 1:** **Ingredient and chemical composition of diets fed during close-up (−21 days to calving), and early lactation**.

**Component**	**Close-up**	**Early-lactation**
**INGREDIENT, % OF DM**		
Alfalfa silage	7.58	4.90
Alfalfa hay	3.50	3.90
Corn silage	39.43	33.13
Wheat straw	8.39	2.64
Cottonseed	–	3.86
Wet brewers grains	6.11	9.42
Ground shelled corn	18.77	22.60
Soy hulls	4.07	3.90
Soybean meal, 48% CP	3.03	5.59
Expeller soybean meal^a^	0.67	3.15
SoyChlor	2.25	–
Blood meal 85% CP	0.63	0.29
Molasses	0.42	–
Urea	–	0.74
Rumen-inert fat^b^	–	1.97
Limestone	2.23	1.56
Salt (Plain)	–	0.26
Ammonium chloride	1.14	–
Dicalcium phosphate	0.31	0.43
Magnesium oxide	0.11	0.13
Magnesium sulfate	1.36	0.26
Sodium bicarbonate	–	0.71
Calcium sulfate	–	0.10
Mineral-vitamin mix^c^	0.17	0.20
Vitamin A^d^	0.03	0.04
Vitamin D^e^	0.02	0.02
Vitamin E^f^	0.36	0.20
**CHEMICAL ANALYSIS**		
Net energy_(lactation)_ (NE_L_), Mcal/kg DM	1.59	1.67
Crude protein (CP), % DM	14.32	18.73
Neutral detergent fiber (NDF), % DM	39.13	35.92
Acid detergent fiber (ADF), % DM	23.94	22.21
Zn, mg/kg DM	83.21	69.00
Mn, mg/kg DM	75.82	70.52
Cu, mg/kg DM	14.41	12.31
Co, mg/kg DM	0.72	0.19

a *SoyPLUS (West Central Soy, Ralston, IA)*.

b *Energy Booster 100 (MSC, Carpentersville, IL)*.

c *Contained a minimum of 4.3% Mg, 8% S, 6.1% K, 2.0% Fe, 3.0% Zn, 3.0% Mn, 5000 mg/kg of Cu, 250 mg/kg of I, 40 mg/kg of Co, 150 mg/kg of Se, 2200 kIU/kg of vitamin A, 660 kIU/kg of vitamin D3, and 7700 IU/kg of vitamin E*.

d *Contained 30,000 kIU/kg*.

e *Contained 5009 kIU/kg*.

f *Contained 44,000 IU/kg*.

### Ruminal digesta sampling

On days −14, −7, 10, 20, and 28 relative to parturition and prior to the morning feeding, after thoroughly mixing of the contents, a grab sample of ruminal digesta was collected via the ruminal cannula from the ventral sac of the rumen. The mixed ruminal content was squeezed through three layers of cheesecloth to separate the ruminal liquid and solid fractions. All samples were immediately placed on ice, transported to the laboratory, and stored at −20°C prior to DNA extraction.

### DNA extraction and quality check

The procedure for extraction of DNA from the mixture of ruminal liquid and solid fractions was adapted from Stevenson and Weimer (2007): 25 g of ruminal solid sample were added into 75 mL of chilled extraction buffer (EB) composed of 100 mM Tris/HCl, 10 mM ethylenediaminetetraacetic acid (EDTA), and 0.15 M NaCl at pH of 8.0. The mixture was blended by polytron (Kinematica Inc., Bohemia, NY, USA) for 2 min and gently centrifuged at 500 × *g* for 15 min at 4°C to remove plant particles while keeping bacterial cells in the suspension. The supernatant was then filtered through a small quantity of pyrex glass wool (Corning Life Science, Corning, NY, USA). The resulting supernatant plus 25 mL of ruminal fluid from the same cow were centrifuged at 10,000 × *g* for 25 min at 4°C. Pellet was harvested and re-suspended in 4 mL ice-cold EB buffer. A 350 μL of the suspension was added into a 1.5 mL microfuge tube containing 0.25 g zirconium/silica beads (0.1 mm diameter; BioSpec Products Inc., Bartlesville, OK, USA). Subsequently, 25 μL of 20% sodium dodecyl sulfate (SDS), and 350 μL equilibrated phenol (pH 8.0; Fisher Sci., Pittsburg, PA, USA) were added to each tube, mixed thoroughly in a bead beater (BioSpec Products Inc., Bartlesville, OK, USA) for 2 min, and incubated at 65°C for 10 min. Tubes were mixed again for an additional 2 min and centrifuged at 12,000 × *g* for 5 min. The supernatants were transferred to new microfuge tubes and extracted twice with 500 μL phenol (pH 8.0), twice with 500 μL phenol/chloroform (pH 8.0; Fisher Sci., Pittsburg, PA, USA), and finally twice with 500 μL chloroform (Fisher Sci., Pittsburg, PA, USA) at 12,000 × *g* for 5 min each. A small amount of EB was added occasionally to the tubes to keep the aqueous volumes above 450 μL. The final supernatant was mixed with a 0.1 volume of 3.0 M sodium acetate (Sigma-Aldrich, St. Louis, MO, USA) and the DNA precipitated with a 0.6 volume of isopropanol (Fisher Sci., Pittsburg, PA, USA). The precipitated DNA pellets were washed twice by adding 1000 μL of 70% ethanol and centrifuged at 12,000 × *g* for 5 min. The supernatant was decanted, the remaining liquid was removed by aspiration, and the DNA pellet dried at room temperature for approximately 20 min. The pellet was resuspended in 100 μL 1X Tris-EDTA Buffer, containing 10 mM Tris-HCl and 1 mM EDTA at pH 8.0. The extracted DNA was stored at −80°C until further analysis. DNA was quantified using a NanoDrop 2000 spectrophotometer (Thermo Fisher Sci., Waltham, MA, USA). DNA samples were normalized to 20 ng/μL, and quality checked by PCR amplification of the 16S rRNA gene using universal primers 27F (5′-GAAGAGTTTGATCATGGCTCAG-3′) and 342R (5′-CTGCTGCCTCCCGTAG-3′) as described by Khafipour et al. (2009). Amplicons were verified by agarose gel electrophoresis.

### Library construction and illumina sequencing

The PCR amplification was targeted to amplify the V4 region of the 16S rRNA gene using modified F515/R806 primers (Caporaso et al., 2012) as described previously (Derakhshani et al., 2016). In brief, the reverse PCR primer was indexed with 12-base Golay barcodes, allowing for multiplexing of samples. For each sample, the PCR reaction was performed in duplicate and contained 1.0 μL of pre-normalized DNA, 1.0 μL of each forward and reverse primer (10 μM), 12 μL HPLC grade water (Fisher Scientific, Ottawa, ON, Canada), and 10 μL of 5 Prime Hot MasterMix (5 Prime Inc., Gaithersburg, MD, USA). Reactions consisted of an initial denaturing step at 94°C for 3 min followed by 35 amplification cycles at 94°C for 45 s, 50°C for 60 s, and 72°C for 90 s, with a final extension step at 72°C for 10 min in an Eppendorf Mastercycler pro (Eppendorf, Hamburg, Germany). Then, PCR products were purified using a ZR-96 DNA Clean-up Kit (ZYMO Research, Irvine, CA, USA) to remove primers, dNTPs and reaction components. The V4 library was generated by pooling 200 ng of each sample and quantified by Picogreen dsDNA (Invitrogen, Burlington, ON, Canada). This was followed by multiple dilution steps using pre-chilled hybridization buffer (HT1; Illumina, San Diego, CA, USA) to bring the pooled amplicons to a final concentration of 5 pM, as determined with a Qubit 2.0 Fluorometer (Life Technologies, Burlington, ON, Canada). Finally, 15% of the PhiX control library was spiked into the amplicon pool to improve the unbalanced and biased base composition, a common characteristic of low-diversity 16S rRNA libraries. Customized sequencing primers for read1 (5′-TATGGTAATTGTGTGCCAGCMGCCGCGGTAA-3′), read2 (5′-AGTCAGTCAGCCGGACTACHVGGGTWTCTAAT-3′) and index read (5′-ATTAGAWACCCBDGTAGTCCGGCTGACTGACT-3′) were synthesized and purified by polyacrylamide gel electrophoresis (Integrated DNA Technologies, Coralville, IA, USA) and added to the MiSeq Reagent Kit v2 (300-cycle; Illumina, San Diego, CA, USA). The 150 bp paired-end sequencing reaction was performed on a MiSeq platform (Illumina) at the Gut Microbiome and Large Animal Biosecurity Laboratories (Department of Animal Science, University of Manitoba, Winnipeg, MB, Canada). The sequencing data are uploaded into the Sequence Read Archive (SRA) of NCBI (http://www.ncbi.nlm.nih.gov/sra) and are accessible through accession number SRR2553327. Metadata used for performing bioinformatics and statistical analyses can be found in Supplementary Table 1.

### Bioinformatics analyses

The PANDAseq assembler (Masella et al., 2012) was used to merge and fix the overlapping paired-end Illumina fastq files. All the sequences with low quality base calling scores as well as those containing uncalled bases (N) in the overlapping region were discarded. The output fastq file was then analyzed by downstream computational pipelines of the open source software package QIIME (Caporaso et al., 2010b). Assembled reads were demultiplexed according to the barcode sequences, chimeric reads were filtered using UCHIME (Edgar et al., 2011) and sequences were assigned to Operational Taxonomic Units (OTU) using the QIIME implementation of UCLUST (Edgar, 2010) at 97% pairwise identity threshold. Taxonomies were assigned to the representative sequence of each OTU using RDP classifier (Wang et al., 2007) and aligned against the Greengenes Core reference database (DeSantis et al., 2006) using PyNAST algorithms (Caporaso et al., 2010a). A phylogenetic tree was built with FastTree 2.1.3. Price et al. (2010) for further comparisons between microbial communities.

Within community diversity (α-diversity) was calculated using QIIME. The alpha rarefaction curve was generated using Chao 1 estimator of species richness (Chao, 1984), with 10 sampling repetitions at each sampling depth. An even depth of 22,000 sequences per sample was used for comparison between richness and diversity indices of ruminal microbial communities. The β-diversity was measured by calculating weighted UniFrac distances (Lozupone and Knight, 2005). Principal coordinate analysis (PCoA) was applied on resulting distance matrices to generate two-dimensional plots using PRIMER v6 software (Warwick and Clarke, 2006). Finally, the open source software PICRUSt (phylogenetic investigation of communities by reconstruction of unobserved states) (Langille et al., 2013) was used to predict functional genes of the classified members of the ruminal microbiota (resulting from reference-based OTU picking against Greengenes database). Predicted genes were then hierarchically clustered and categorized using the KEGG (Kanehisa and Goto, 2000) orthologs (KOs) and pathways (levels 1–2).

### Statistical analysis

Statistical analyses of phylogenetic data were performed as described by Li et al. (2012). In brief, partial least squares discriminant analysis (PLS-DA; SIMCA P+ 13.0, Umetrics, Umea, Sweden) was performed on genus data (relative abundances) to identify the effects of sampling time-points. The PLS-DA is a particular case of partial least squares regression analysis in which Y is a set of variables describing the categories of a categorical variable on X. In this case, X variables were the relative abundances of bacterial genera and Y variables were observations of different days pre- or postpartum versus each other. The weights for the X-variables, denoted “w,” indicate the importance of these variables, how much they “in a relative sense” participate in the modeling of Y. The weights for the Y-variables, denoted by “c,” indicate which Y-variables are modeled in the respective PLS model dimensions. For this analysis, data were scaled using Unit Variance in SIMCA. Cross-validation was then performed to determine the number of significant PLS components and a permutation test was conducted to validate the model. To avoid over parameterization of the model, variable influence on projection value (VIP) was estimated for each genus and genera with VIP < 0.50 were removed from the final model (Pérez-Enciso and Tenenhaus, 2003; Verhulst et al., 2011). An R^2^ estimate was then used to evaluate the goodness of fit and a Q^2^ estimate was used to evaluate the predictive value of the model. The PLS-regression coefficients were used to identify genera that were most characteristic of each sampling time-point and the results were visualized by PLS-DA loading scatters plots, which are superimpositions of the w (weight of the X-variables: bacterial genera) and c [weights of the Y-variables (classes): days relative to calving] plots, for the first and second components of the PLS-DA model. X-variables situated in the vicinity of the Y-variables (class IDs: days relative to calving) have the highest discriminatory power between the classes.

The UNIVARIATE procedure of SAS (SAS 9.3, 2012) was used to test the normality of residuals for α-diversity measurements. Non-normally distributed data were subjected to either log or box-cox power transformation and then used to assess effects of sampling time-point, using the MIXED procedure of SAS. The null hypotheses being tested was that the diversity of ruminal microbial communities of cows collected at different time points during the periparturient period are similar. Effect of time of sampling was considered as the fixed factor, whereas the effect of individual cows was considered as the random factor in all comparisons. All pairwise comparisons among the groups were tested using Tukey's studentized range adjustment, and statistical significance was declared at a *P*-value < 0.05. Trends are discussed at *P*-value < 0.10.

Unsupervised clustering analysis was performed to relate the proportion of abundant bacterial genera to the clustering pattern of samples: bacterial genera were filtered based on their relative abundance (cutoff value of >0.05% of community). The resulting relative abundance table was normalized (values divided by the Euclidean length of the row vector) to correct for compositionality and also assist heatmap-visualization of differentially abundant genera. Bray–Curtis measures of dissimilarity were calculated using R “vegan” package (Oksanen et al., 2007) and the resulting matrix was subjected to unsupervised hierarchical clustering using R “dendextend” package (Galili, 2015) and visualized over the heatmap of abundance matrix using R “complexheatmap” package (Gu et al., 2016).

The relationship between abundant ruminal bacteria and physiological parameters was explored as described by Jami et al. (2014). In brief, the PAST software (Hammer et al., 2012) was used to perform the Spearman's rank correlation coefficient test, and the resulting correlation matrix was visualized in heatmap format generated by the corrplot package of R (Wei, 2010). All pairwise correlations with a *P*-value < 0.05 were considered significant. Furthermore, statistical analysis on the proportion of functional genes and pathways was performed using the linear discriminant analysis (LDA) effect size (LEfSe) (Segata et al., 2011), a software principally developed to discover metagenomics biomarkers. Analyses included the non-parametric factorial Kruskal-Wallis (KW) sum rank test (Kruskal and Wallis, 1952), followed by LDA to estimate the effect size of each differentially abundant feature. The threshold on the logarithmic LDA score for discriminative features was set at 2.0, so that features with at least 100-fold shift were considered significant.

## Results

### High-throughput 16S rRNA gene sequencing of ruminal microbial communities

**Figure 1:**
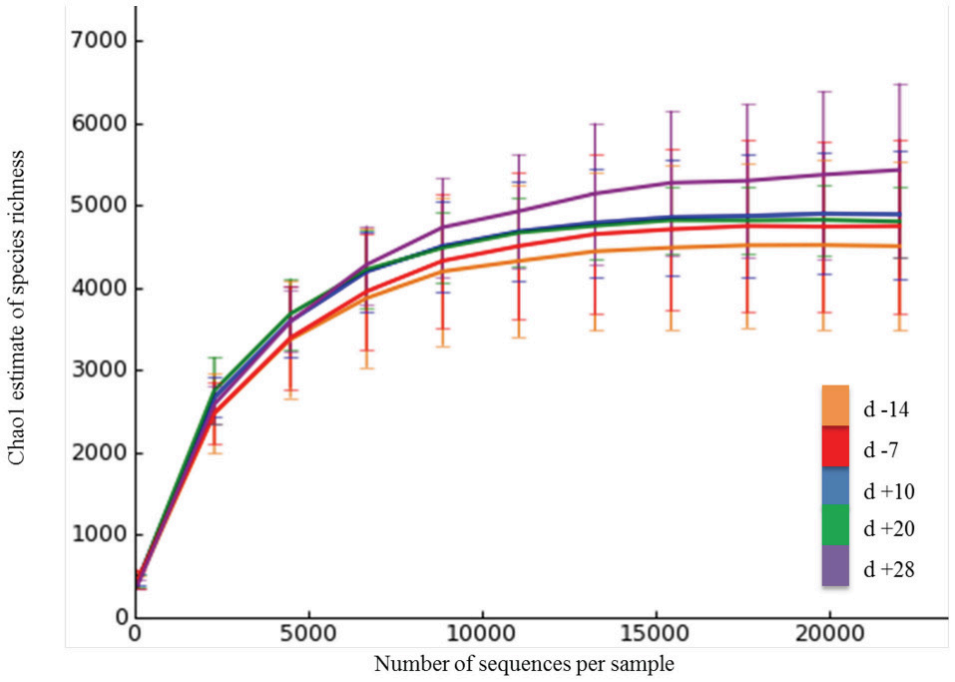
**Rarefaction analysis for the observed species**. The rarefaction curve was generated using Chao1 richness estimator. Samples were rarified at an even depth of 22,000 sequences per sample. Color codes denote samples collected at different days relative to calving. Error bars denote the 95% confidence intervals.

On average, Illumina paired-end sequencing generated 34,067 of high-quality sequences per sample, with median sequencing length of 253nt covering the full length of the V4 hypervariable region of the 16S rRNA gene. The OTU clustering at 97% similarity threshold resulted in a Good's nonparametric coverage estimate (Good, 1953) of 93.22% across all samples (Table 2). At an even depth of 22,000 sequences per sample, the number of observed species across samples increased numerically postpartum. Similarly, an increasing trend in the Chao1 estimates of species richness was observed postpartum, which began to plateau at around 20,000 sequences per sample (Figure 1). Overall, no significant change was observed in Shannon and Simpson indices of diversity in ruminal microbial communities during the peripartal period (Table 2).

**Table 2:** **Summary statistics for diversity indices observed in ruminal microbial communities during the peripartal period**.

**Day**	**Mean results for indicated variable**					
	**Number of sequences per sample**	**Observed number of species**	**Goods-Coverage (%)**	**Richness^a^**	**Diversity^b^**	
				**Chao1**	**Shannon**	**Simpson**
d −14	22,000	3449	93.83	4508	9.55	0.99
d −7	22,000	3510	93.34	4752	9.50	0.99
d 10	22,000	3615	93.13	4894	9.60	0.99
d 20	22,000	3710	93.47	4807	9.72	0.99
d 28	22,000	3866	92.28	5439	9.54	0.99
SED^c^	–	267	0.83	503	0.19	<0.01
*P*-value	–	0.85	0.48	0.50	0.82	0.22

a *Based on Chao1 estimator of species richness*.

b *Based on Shannon and Simpson diversity estimators*.

c *SED, standard error of difference between least square means of treatments*.

Taxonomic classification of clustered OTUs revealed the presence of 16 bacterial phyla (Table 3). While the majority of OTUs were identified at the genus (g.) or species levels, some were only classified at the phylum (p.), class (c.), order (o.), or family (f.) level. Abundant phyla (above 1% of community) included Firmicutes, Bacteroidetes and Tenericutes, whereas Actinobacteria, Cloroflexi, Proteobacteria, SR1, Verrucomicrobia were in medium-abundance (between 0.1 and 1% of community) and Armatimonadetes, Cyanobacteria, Fibrobacteres, Planctomycetes, Spirochaetes, Synergistetes, TM7 and WPS-2 were in low abundance (below 0.1% of community). The proportions of Tenericutes, Cloroflexi, and Verrucomicrobia phyla were higher during the prepartum (*P* < 0.05), and Actinobacteria during the postpartum.

**Table 3:** **Relative abundances of bacterial phyla in the ruminal digesta in five different time points pre- and postpartum (d −14, d −7, d 10, d 20, and d 28) during the peripartal period**.

**Phyla**	**Mean percentage of phyla**					**SED^1^**	***P*****-value**	**Contrast**
	**Prepartum**		**Postpartum**					**Prepartum vs. Postpartum**
	**d −14**	**d −7**	**d 10**	**d 20**	**d 28**			
	**Above 1% of community**							
Bacteroidetes	15.44	13.75	16.16	17.87	20.92	3.54	0.40	0.11
Firmicutes	78.84	81.77	80.02	78.01	75.50	3.83	0.63	0.33
Tenericutes	1.65^a^	1.52^a^^b^	1.11^a^^b^^c^	0.83^b^^c^	0.71^c^	0.35	0.06	0.006
	**Between 0.1 and 1% of community**							
Actinobacteria	0.14^a^^b^	0.12^a^	0.18^a^^b^	0.21^b^	0.23^b^	0.07	0.10^*^	0.009^*^
Chloroflexi	1.11^a^	0.68^a^^b^	0.46^b^	0.58^b^	0.21^b^	0.26	0.03	0.009
Proteobacteria	0.59	0.31	0.30	0.85	0.81	0.36	0.45	0.40
SR1	0.09	0.08	0.18	0.09	0.06	0.04	0.09^*^	0.47^*^
Verrucomicrobia	0.33^a^	0.21^a^^b^	0.12^a^^b^	0.11^b^	0.09^b^	0.07	0.01^*^	0.001^*^
	**Below 0.1% of community**							
Armatimonadetes	0.008	0.006	0.001	0.002	0.003	0.002	0.47^*^	0.88^*^
Cyanobacteria	0.01	0.01	0.02	0.06	0.05	0.02	0.49^*^	0.48^*^
Fibrobacteres	0.19	0.14	0.02	0.01	0.02	0.14	0.89^*^	0.60^*^
Planctomycetes	0.03	0.03	0.03	0.03	0.01	0.008	0.45	0.21
Spirochaetes	0.14	0.08	0.01	0.02	0.03	0.08	0.42^*^	0.26^*^
Synergistetes	0.04	0.02	0.02	0.03	0.05	0.02	0.27^*^	0.17^*^
TM7	0.004	0.003	0.003	0.004	0.004	0.002	0.86^*^	0.79^*^
WPS-2	0.018	0.015	0.011	0.007	0.001	0.007	0.32^*^	0.07^*^

a, b, c *Means within a row with different superscripts differ (P < 0.05)*.

* *Statistical analyses were conducted on log_10_-transformed data*.

1 *SED, standard error of difference between least square means of treatments*.

### Structural shifts in ruminal microbial communities during the peripartal period

**Figure 2:**
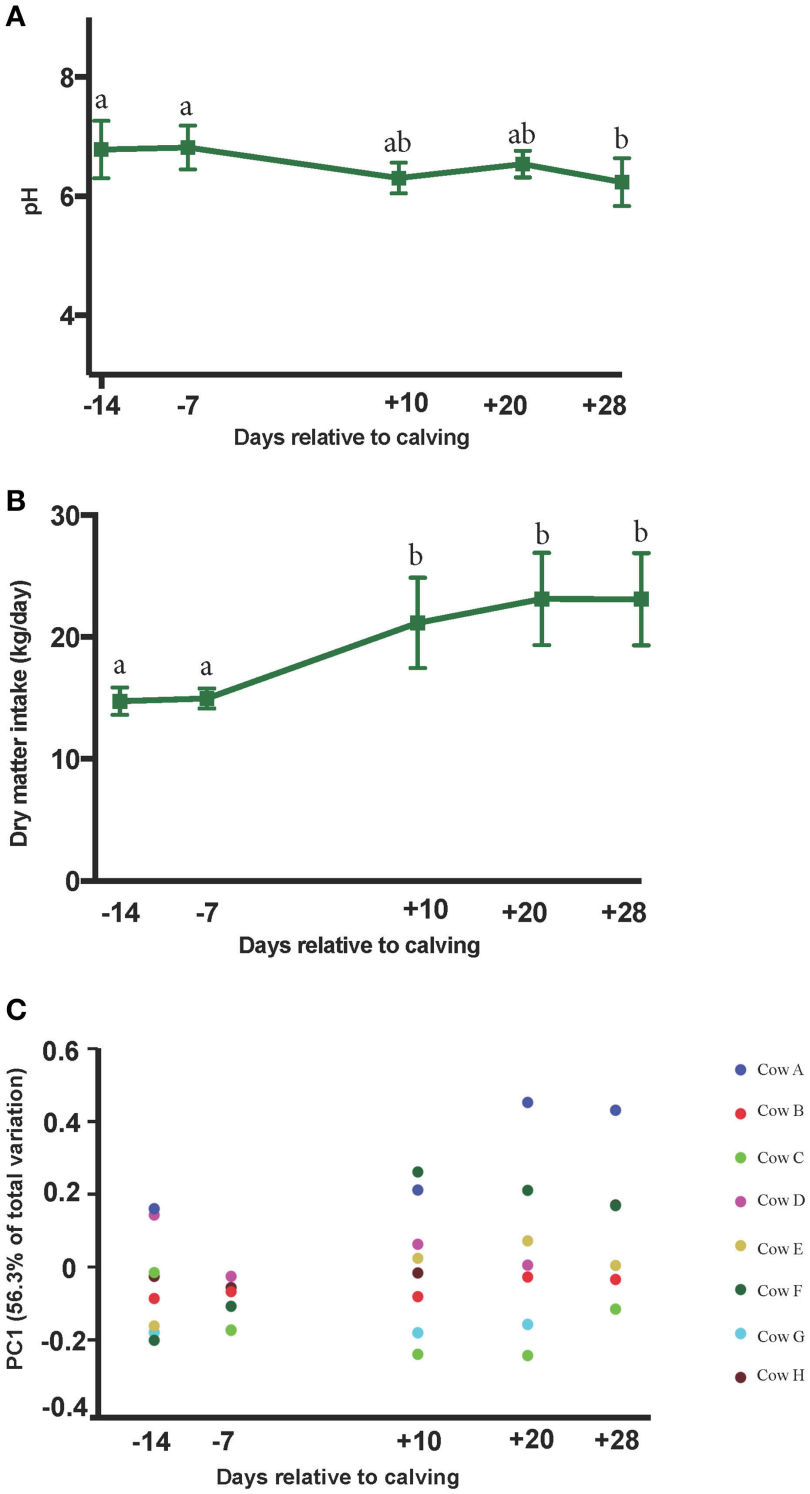
**Peripartal shifts in dry matter intake, rumen pH and the ruminal microbial community**. Changes in the **(A)** rumen pH, and **(B)** dry matter intake are plotted against sampling time points. Superscripts denote significant differences (*P* < 0.05) between the means and error bars denote the 95% confidence intervals. **(C)** Weighted UniFrac distances of ruminal microbial communities were minimal prepartum and increased postpartum. Colored circles are indicative of individual cows. Y axis shows the percentage of variation among communities explained by first principal component (PC1).

The PERMANOVA analyses of weighted UniFrac distances among microbial communities revealed distinct clustering patterns between pre- and postpartal ruminal microbiota (*P* = 0.01). Pairwise comparisons included d −14 vs. d 28 (*P* = 0.03), d −14 vs. d 20 (*P* = 0.16), d −14 vs. d 10 (*P* = 0.15), d −7 vs. d 28 (*P* = 0.05), and d −14 vs. d −7 (*P* = 0.51; Supplementary Figure 1). Plotting weighted UniFrac distances over time indicated smaller variations in the ruminal microbiota among cows in the prepartum period, but the differences gradually increased in the postpartum. A similar trend was observed for DMI during the same period of time. The average daily DMI increased significantly from 14.7 kg on day −14 to 23.1 kg on day 28, while changes in the ruminal pH values were modest (ranging from 6.78 to 6.23 on days −14 and 28, respectively; Figures 2A–C).

**Figure 3:**
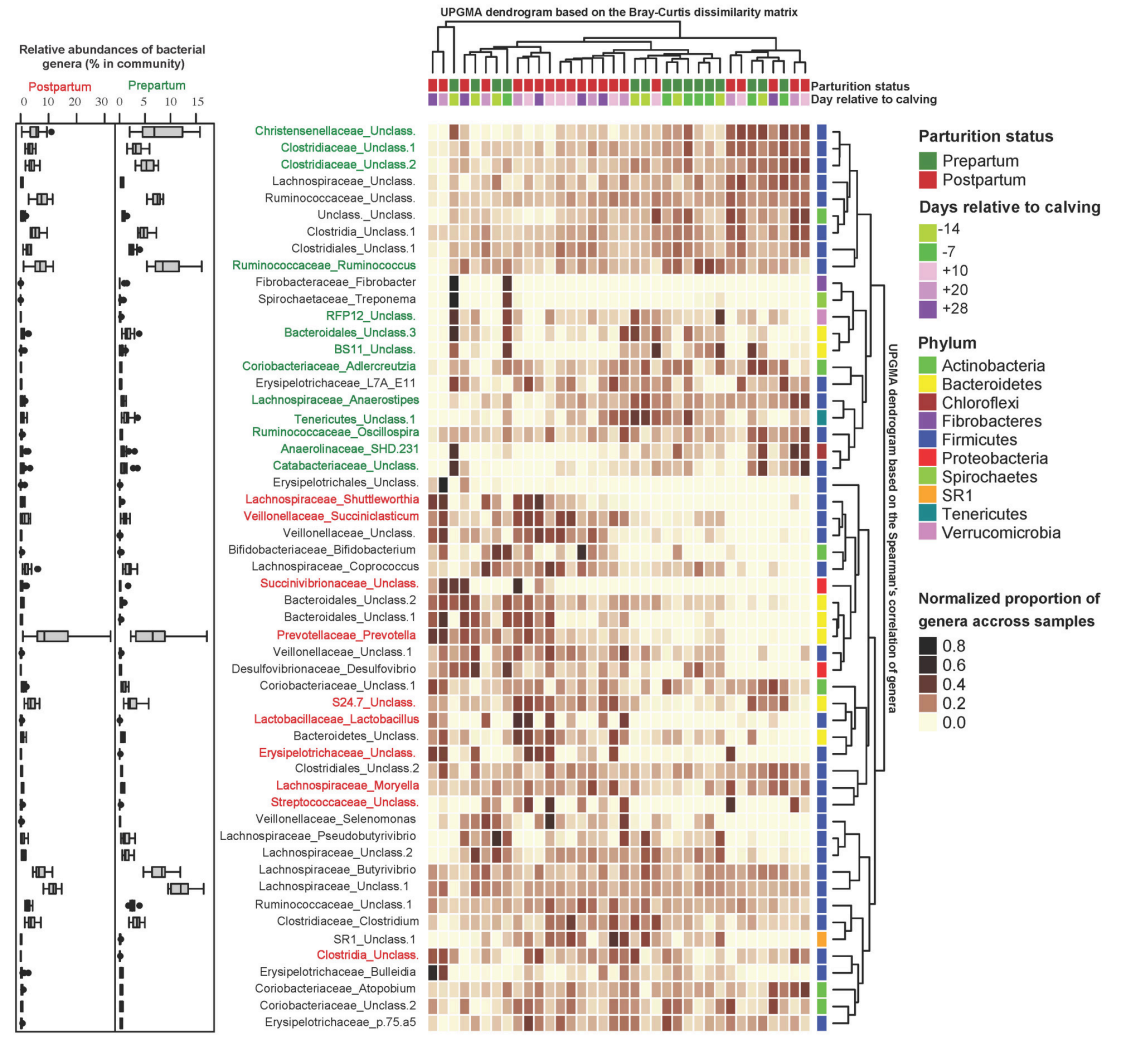
**Unsupervised cluster analysis of peripartal ruminal microbial communities**. Columns correspond to samples and rows correspond to abundant genera (>0.05% of community). The “Normalized proportion” key relates colors to the normalized proportions of genera across samples (relative abundance of each genus divided by the Euclidean length of the row vector). The top dendogram depicts how samples are clustered based on their Bray–Curtis dissimilarities [using unweighted pair group method with arithmetic averaging (UPGMA)]. The right dendogram depicts how genera correlate (co-occur) with each other based on their Spearman's correlation coefficient. The “Phylum” key relates the right annotations to the corresponding phylum of each genus. The “Parturition status” and “Days relative to calving” keys relate samples to their corresponding sampling time points. The left box-plots depict the average relative abundance of genera during the pre- (right) and post-partum (left) periods. Color codes have been used to depict the association of genera with parturition status (identified using PLS-DA as detailed in Supplementary Figure 2): genera highlighted in green depict significant positive association with days −14 and −7, and genera highlighted in red depict significant positive association with days 10, 20, and 28.

Figure 3 depicts the results of unsupervised clustering analysis (Bray-Curtis measures of dissimilarity) based on the proportion of abundant bacterial genera. Despite large inter-animal differences that existed between the proportions of abundant genera, in general, pre- and postpartal ruminal microbiota tended to cluster separately. PLS-DA was performed to explore the association of bacterial genera with sampling time points (pairwise comparisons included d −7 vs. d 28, d −14 vs. d 28, d −14 vs. d 20, d −14 vs. d 10, d 10 vs. d 28, and d 20 vs. d 28; Supplementary Figures 2A–F). In summary, members of the p. Firmicutes (g. *Ruminococcus, Dehalobacterium, Adlercreutzia, Christensenellaceae*), p. Bacteroidetes (f. BS11), p. Tenericutes (c. Mollicutes and o. RF39), p. Verrucomicrobia (f. WCHB1-25), and p. Chloroflexi (g. SHD-231) were enriched on d −7, whereas several bacterial taxa within p. Firmicutes (g. *Streptococcus, Lactobacillus* and *Shuttleworthia*), p. Bacteroidetes (g. *Prevotella* and *Bacteroides*), and p. Proteobacteria (g. *Succinivibrio*) were more abundant at d 28 compared with d −7.

### Relationship of the ruminal microbiota with production parameters

**Figure 4:**
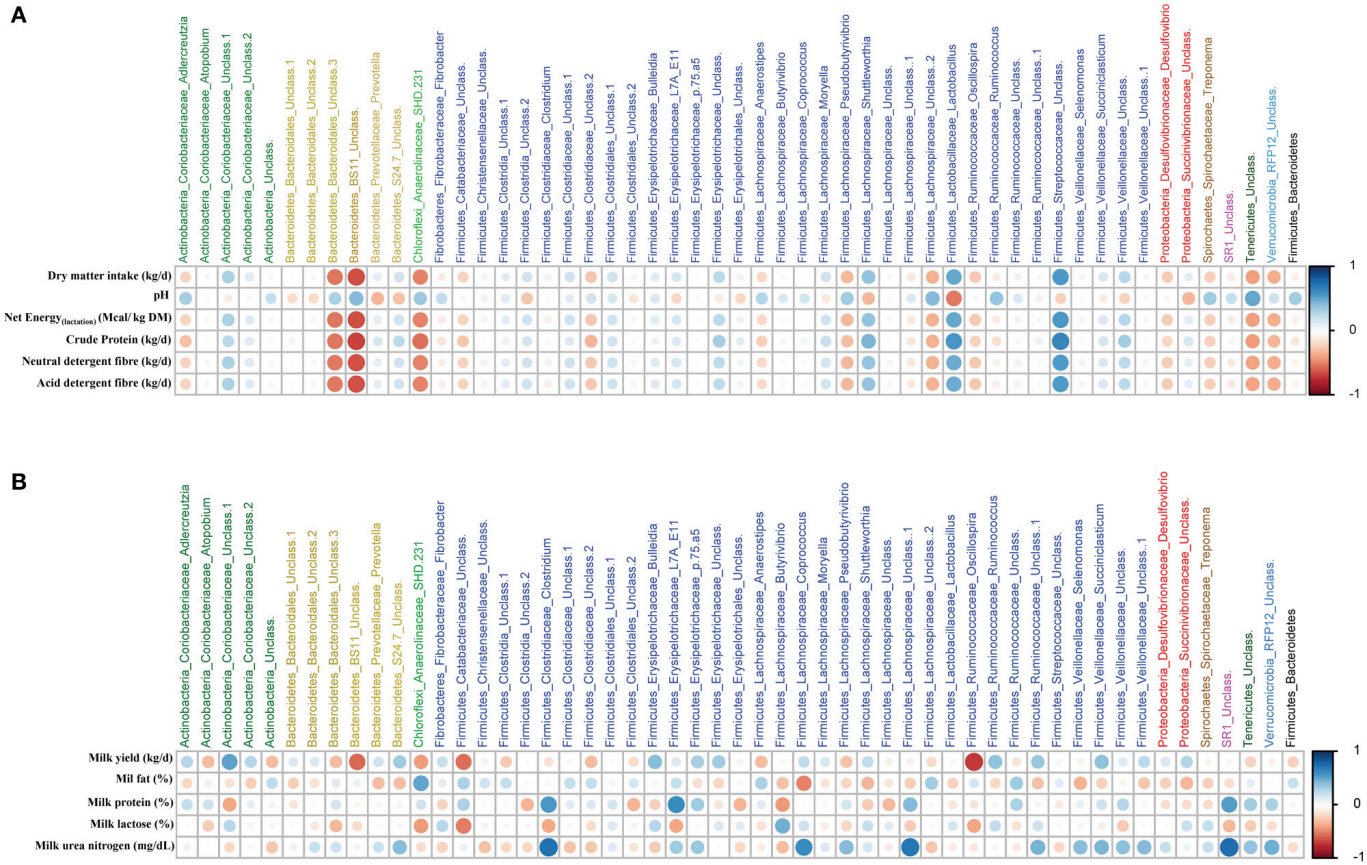
**Relationships among ruminal microbiota and production parameters**. The correlation matrix was based on the Spearman's rank correlation coefficient of the abundant ruminal bacterial genera (>0.05% of community) with **(A)** pre- and postpartum feed intake and chemical composition of the diet, and **(B)** postpartum milk yield and composition. The strength of the correlation between each pair of variables is indicated by diameter and color intensity of the circles. A color code of dark blue indicates a positive correlation coefficient close to +1 and a color code of dark red indicates a negative correlation coefficient close to −1. The last column on the right side of each matrix is also based on the correlation of the Firmucures:Bacteroidetes ratio with production parameters.

Based on Spearman's correlation coefficients, significant correlations (*P* < 0.05) were detected between ruminal microbiota, feed intake and diet chemical composition (Figure 4A), and milk yield and milk composition (Figure 4B). One group of ruminal microbiota including unclassified Bacteroidales and BS11 (within Bacteroidetes), SHD-231 (within Chloroflexi), unclassified Lachnospiraceae (within Firmicutes), and unclassified Tenericutes had strong negative correlations with daily intakes of DMI, crude protein (CP), neutral detergent fiber (NDF), and acid detergent fiber (ADF), whereas they were positively correlated with ruminal pH. On the other hand, genera *Lactobacillus, Shuttleworthia*, and unclassified Streptococcaceae (all within Firmicutes) were negatively correlated with the aforementioned parameters. *Lactobacillus* and *Prevotella* were the only genera with significant negative correlations with ruminal pH. With regards to milk production, unclassified Coriobacteriaceae (within Actinobacteria) was the only genus with a positive correlation with milk yield, whereas, unclassified BS11, unclassified Catabacteriaceae and g. *Oscillospira* (both within Firmicutes) were negatively correlated with milk yield. Several members of Firmicutes (including *Clostridium, Coprococcus*, and unclassified Lachnospiraceae) were positively correlated with milk urea nitrogen (MUN). *Clostridium* and unclassified Lachnospiraceae, along with unclassified Erysipelotrichaceae, were also positively correlated with milk protein percentage. See Supplementary Table 2 for the summary statistics of Spearman's correlations of ruminal microbiota with production parameters and Supplementary Figure 3 for postpartal distributions/comparisons of milk yield and milk composition.

### Metagenomics imputation

**Figure 5:**
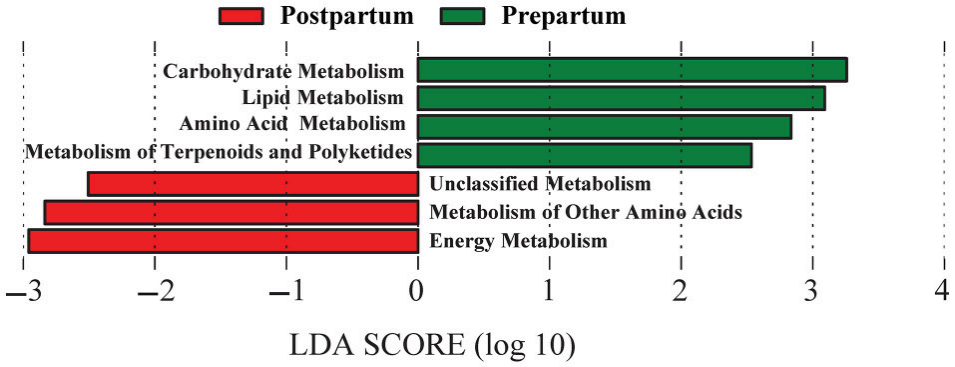
**Differentiation of predicted functional metagenomes of pre and postpartal ruminal microbial communities**. Linear discriminant analysis (LDA) was performed to identify significant changes in the proportion of reconstructed functional pathways (obtained from PICRUSt predictive algorithms at KEGG level 2). Analysis was performed using LEfSe, a metagenome analysis approach which performs LDA following Wilcoxon Mann-Whitney test to assess the effect size of each differentially abundant variable. Color code represents the class of treatment (Red indicates variables that were detected as significantly more abundant during the postpartal period and green indicates variables that were detected as significantly more abundant during the prepartal period).

Reference-based OTU picking resulted in 74% of the sequencing reads being mapped to the Greengenes database, which were subsequently used for prediction of the functional genes and assigning KEGG orthologs and their corresponding pathways (at level 2). Genes associated with carbohydrate, lipid, amino acid, and terpenoids and polyketides metabolism pathways were overrepresented (*P* < 0.05; change >100-fold) in prepartal-associated ruminal microbiota, whereas genes associated with energy metabolism and other amino acids metabolism were enriched in postpartal ruminal microbiota (Figure 5).

## Discussion

In the current study, we used Illumina deep-sequencing technology to characterize the changes of ruminal microbiota during the periparturient period. During this period, high-producing dairy cows commonly experience a gradual decline in voluntary DMI (Marquardt et al., 1977; Bell, 1995; Grummer et al., 2004). Prepartal provision of energy-dense diets is, therefore, a common practice aimed to ease the metabolic transition from late-pregnancy to early lactation, and also to facilitate ruminal microorganisms to adapt to fermenting higher-grain/higher-energy lactation diets after calving (Hayirli et al., 2002; Grummer et al., 2004). Our findings indicated that fluctuations in the intake and chemical composition of the diet were the main factors responsible for differences in composition and functional properties of pre- and postpartal ruminal microbiota. In addition, correlations among the proportions of ruminal microbiota and production parameters, such as milk yield and milk composition, underscored the metabolic potential of the ruminal microbial community.

Health of the rumen is partly associated with proper richness, diversity and stability of the ruminal microbiome (Khafipour et al., 2016). In this study, although the average Chao1 estimate of richness tended to increase postpartum, other alpha-diversity parameters of ruminal microbiota did not change during the transition period. However, it is noteworthy that based on UniFrac distances, we observed that individual variation in the composition of ruminal microbiota tended to be lowest on day −7 and started to increase postpartum. The amount of dietary intake and its chemical composition are, in general, the most important factors in determining the diversity and functional properties of the gut microbiota (Turnbaugh et al., 2009a; Spor et al., 2011). In peripartal dairy cows, the “close-up” diet usually contains the same higher-energy ingredients (e.g., corn grain) as the lactating diet to allow ruminal microbes enough time to adapt to the dietary changes that occur after calving (Overton and Waldron, 2004; Jouany, 2006). In the present work, temporal dynamics of the beta-diversity of microbial communities followed a similar trend to that of the inter-animal differences in DMI, suggesting that increased dietary intake, and consequently higher nutrient availability, plays a central role in shaping more heterogeneous profiles of ruminal microbiota in the postpartum period.

Based on weighted UniFrac distances, we also observed a significant impact of the host animal on the clustering pattern of ruminal microbiota. This inter-animal variability has been extensively reported (Li et al., 2009; Weimer et al., 2010; Jami and Mizrahi, 2012; Jami et al., 2014), highlighting the impact of host genotype and phenotype on the development of the ruminal microbial ecosystem. Notwithstanding, we observed several members of the ruminal microbiota that were dominant across all cows and sample collection times. The existence of such a “core microbiome” is thought to be crucial for maintaining the “functional redundancy” of the ruminal ecosystem. This redundancy is a characteristic defined by the ability of a core microbiome to maintain its major functional properties regardless of phylogenetic fluctuations, and usually occurs when several members of a microbiome share similar functions (Shade and Handelsman, 2012).

Similar to previous studies (Fernando et al., 2010; Mao et al., 2013; Jami et al., 2014; Pitta et al., 2014a), we also observed that Firmicutes and Bacteroidetes were the predominant bacterial phyla in the ruminal ecosystem. The Firmicutes-to-Bacteroidetes ratio has traditionally been considered as a biomarker for metabolic potential of the gut microbiota and is known to modulate host metabolism, physiology, and health (Mariat et al., 2009; Tilg and Kaser, 2011; Clemente et al., 2012). In our study, the temporal shifts in the abundance of these two main phyla were not statistically different. Our results are similar to the findings of Lima et al. (2015), but inconsistent with Pitta et al. (2014a). However, comparisons across microbiome studies should be exercised with extreme caution because a number of biotic and/or abiotic factors unrelated to the physiological responses of the cows to the dietary interventions could be at play. One aspect of our study that requires further attention is the DNA extraction approach (Stevenson and Weimer, 2007). Although this extraction protocol has been optimized to maximize the separation of particle-associated microbiota from plant fibers, the absence of a direct bead-beating step performed on the fiber particles may have caused an underrepresentation of the sequences that belong to certain lineages of fiber-degrading biofilm, i.e., bacteria that are firmly attached to the plant particles and are encapsulated by self-produced extracellular polymeric substances.

Notwithstanding, we observed that the genera *Prevotella* and *Bacteroides*—which collectively constituted the vast majority of OTUs assigned to phylum Bacteroidetes—were positively associated with the postpartal ruminal ecosystem, whereas, genera *Ruminococcus, Butyrivibrio*, and family Christensenellaceae—members of Firmicutes—were enriched in the prepartal ruminal ecosystem. Current understanding of the role of Christensenellaceae in ruminal metabolism is limited. Lima et al. (2015) also reported this family as a dominant member of the prepartal core microbiome. *Ruminococcus* and *Butyrivibrio* include major fibrolytic rumen dwellers and their replacement by *Prevotella* spp.—which are primarily known for their amylolytic and proteolytic properties—has been extensively reported during adaptations of the ruminal microbiota to energy-dense diets (Callaway et al., 2010; Fernando et al., 2010; Pitta et al., 2014a,b).

Another noteworthy feature of postpartal ruminal microbiota was the overrepresentation of genera *Streptococcus* and *Lactobacillus*. These genera consist of starch-utilizer and lactate-producer species, and their ability to proliferate in response to feeding high-grain diets has been reported previously (Russell and Hino, 1985; Ding et al., 2014). The mechanism underlying this bacterial shift can be traced back to the ability of these genera to tolerate high concentrations of ruminal lactate. Such high lactate concentrations in the ruminal ecosystem typically occur during adaptations to high-grain diets, and is known to suppress the growth of pH-sensitive cellulolytic bacteria such as *Megasphaera elsdenii* and *Selenomonas ruminantium*. As a result, the ruminal environment becomes more favorable for the growth of low-pH tolerant bacteria such as *Streptococcus bovis* and *Lactobacilli* spp. (Russell et al., 1981; Jouany, 2006). In the present study, *Lactobacillus* was the only bacterial genus with a strong negative correlation with ruminal pH. In addition, genera *Lactobacillus* and unclassified Streptococcaceae had strong positive correlations with other dietary parameters, including DMI and NE_*L*_. This highlights their contribution to the metabolism of higher dietary starch that is fed during the postpartal period.

In the present study, the shifts in the composition of ruminal microbiota that occurred during the prepartum period were not limited to the bacterial lineages from Firmicutes and Bacteroidetes. Genera belonging to the phylum Proteobacteria, including unclassified Succinivibrionaceae and *Succinivibrio*, were also enriched in the postpartal period. Petri et al. (2013) previously reported Succinivibrionaceae as a core member of the high-grain-associated ruminal microbiota. Several strains within the genus *Succinivibrio* with the ability to ferment starch and produce large amounts of acetic and succinic acids have been isolated from ruminal fluid (Bryant et al., 1958; Santos and Thompson, 2014). We speculate that this bacterial lineage plays an important role in ruminal fermentation of carbohydrates during adaptations to lactation diets. On the other hand, genus *SHD-231* was overrepresented in the prepartal ruminal ecosystem. The metabolic role of this bacterial lineage is poorly understood. In the present work, more than 95% of the OTUs that were classified under the phylum Chloroflexi belonged to the genus *SHD-231*, which could account for the significant postpartal decrease observed in the proportion of this phylum. This is in agreement with the study conducted by Liu et al. (2014) reporting a significant increase in the proportion of Chloroflexi in the cecal microbial communities of goats fed high-grain diets. In contrast, others reported opposite responses of Chloroflexi in animals fed energy-dense diets (Hook et al., 2011; Mao et al., 2016).

Another notable finding of the current study were the positive correlations between genus *Clostridium*, milk protein content and MUN. It is known that microbial–derived amino acids can constitute up to two-thirds of the amino acids absorbed from the ruminant small intestine (Council, 1992; Dewhurst et al., 2000). MUN is also closely related to blood urea N and it can be used to estimate ruminal protein metabolism/synthesis and N utilization efficiency (Roseler et al., 1993; Jonker et al., 1998). Clostridial species isolated from ruminal contents have been characterized as potent proteolytic and/or ammonia hyper-producing bacteria (Attwood et al., 1996; McSweeney et al., 1999). Our results underscore the potential contribution of genus *Clostridium*, in general, to ruminal protein metabolism/synthesis.

Lastly, based on predicted functional metagenomics data, we observed that the proportions of carbohydrate, lipid, and amino acid metabolism pathways are overrepresented during the prepartal period. This was surprising because our initial expectation was that, due to the increase in DMI, and consequently higher availability of carbohydrates, lipids, and CP, the relative abundance of the aforementioned pathways would be higher in the postpartal ruminal microbiome. This observation may be explained by the prepartal shift from far-off to close-up diet, which is more nutrient dense and is assumed to facilitate a successful transition into the lactation diet by compensating for the decrease in DMI (Overton and Waldron, 2004; Jouany, 2006). We speculate that compared with the postpartal period, the close-up period is characterized by a more competitive ruminal ecosystem which selectively favors the growth of bacterial lineages that are flexible in harvesting energy, such as certain members of Bacteroidetes and Firmicutes that possess large repertoires of genes involved in carbohydrate metabolism (Turnbaugh et al., 2009b). Therefore, the proportion of the genes associated with these lineages of bacteria would be higher in prepartal compared with the less competitive ruminal ecosystem of the postpartal period. However, as expected, we observed that during the postpartal period, the overall energy metabolism capacity of the microbiota increased concomitant with the surge of voluntary DMI and availability of a more nutrient-dense diet. Together, our findings suggest that the two-tiered feeding management achieved its goal by acclimating the ruminal microbiome of late-pregnant cows, and prepared them for harvesting energy efficiently from both close-up and lactation diets.

## Conclusions

Overall, our findings demonstrated distinct ruminal microbiota profiles the pre and postpartum period, underscoring a dynamic response of this complex network of microorganisms to the amount and chemical composition of the dietary intake, and its association with changes in production parameters such as milk yield and milk composition. Concomitant with lower voluntary prepartal feed intake, we observed strikingly lower inter-animal variation in the composition of the ruminal microbiota. Genera *Ruminococcus* and *Butyrivibrio*, which consist of major fibrolytic rumen dwellers, were overrepresented in prepartal ruminal ecosystem. In contrast, increased postpartal DMI was associated with enrichment of bacterial lineages such as *Prevotella, Streptococcus*, and *Lactobacillus*, which mainly consisted of amylolytic and proteolytic species. These, together with the postpartal enrichment of energy metabolism pathways, suggested a degree of acclimatization of the ruminal microbiota to the nutrient-dense lactation diet.

## Author contributions

JL designed the study. FC conducted the experiment. HD performed lab analyses. HD and EK developed the bioinformatics and statistical models. HD analyzed the data. HD, HT, JP, JR, and EK wrote the manuscript.

## Funding

Animal experiment was supported by Hatch funds under project ILLU-538–914, National Institute of Food and Agriculture, Washington, DC, USA. Microbiome research was funded by University of Manitoba Internal Grant.

### Conflict of interest statement

The authors declare that the research was conducted in the absence of any commercial or financial relationships that could be construed as a potential conflict of interest.
