# Supplementary material for: Linking Peripartal Dynamics of Ruminal Microbiota to Dietary Changes and Production Parameters
Source: Front Microbiol. 2017 Jan 12;7:2143. doi: 10.3389/fmicb.2016.02143 (PMC5226935; doi:10.3389/fmicb.2016.02143)
Supplement: Supplementary file 3 [file Image1.PDF]

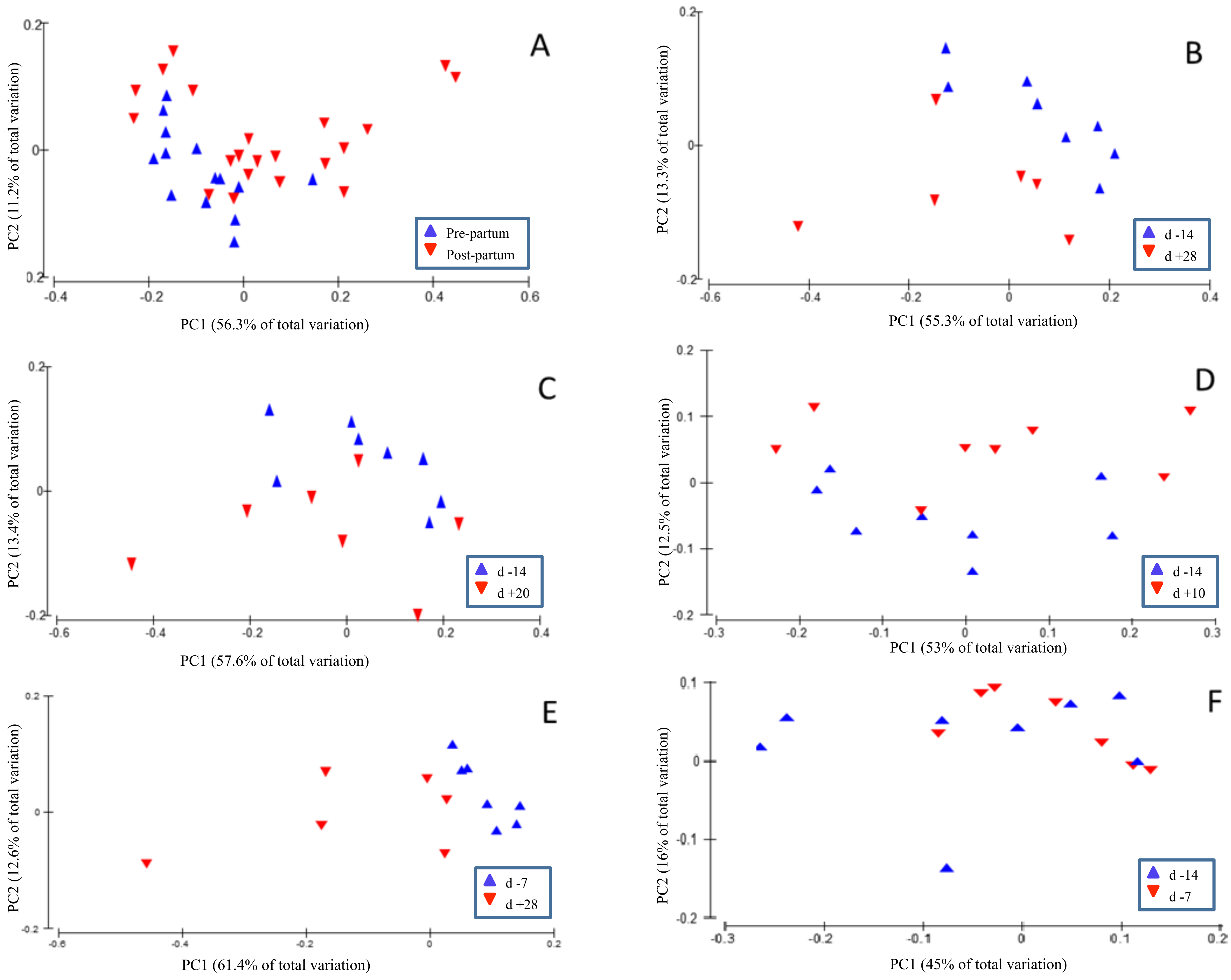

Supplemenray figure 1. Principal coordinate analysis (PCoA) of weighted UniFrac distances between microbial communities of ruminal samples. Comparisons include A) pre vs. postpartal ($P = 0.01$), B) d -14 vs. d 28 ($P = 0.03$), C) d -14 vs. d 20 ($P = 0.16$), D) d -14 vs. d 10 ($P = 0.15$), E) d -7 vs. d 28 ($P = 0.05$), and F) d -14 vs. d -7 ($P = 0.51$). P-value for each comparison was obtained from PERMANOVA and considered significant at $P < 0.05$.
