# Supplementary material for: Linking Peripartal Dynamics of Ruminal Microbiota to Dietary Changes and Production Parameters
Source: Front Microbiol. 2017 Jan 12;7:2143. doi: 10.3389/fmicb.2016.02143 (PMC5226935; doi:10.3389/fmicb.2016.02143)
Supplement: Supplementary file 4 [file Image2.PDF]

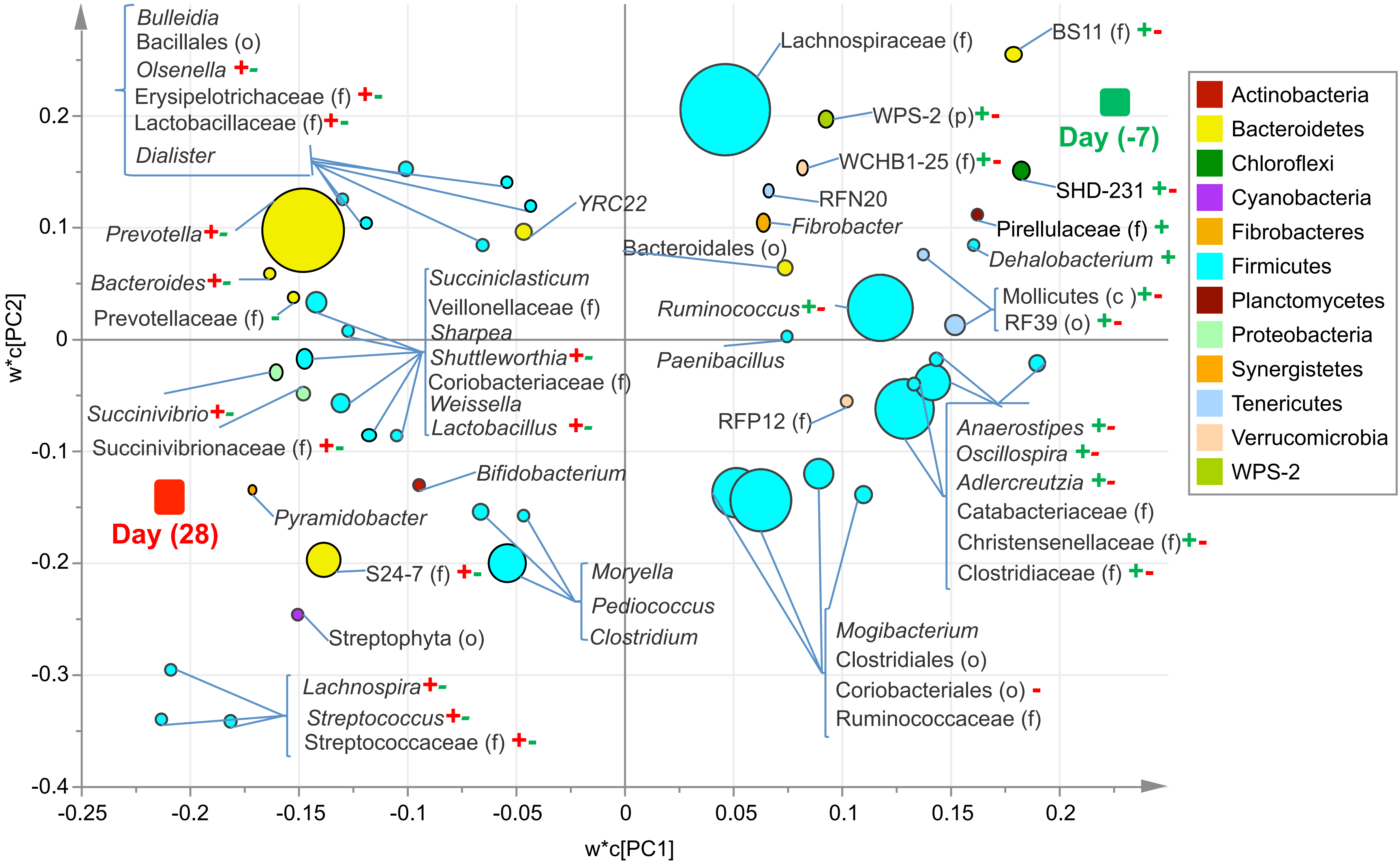

Supplementary figure 2.a. **Partial least square discriminant analysis (PLS-DA) of ruminal microbial communities comparing d -7 vs. d 28.** Bacterial genera are plotted according to their association with d -7 and d 28, colored based on their corresponding phyla, and sized based on their relative abundances. (+) or (-) indicate genera had a positive or negative association with each time point. Some genera could only be affiliated to phylum (P), order (O), or family (F) levels. The R^2 (0.98) and Q^2 (0.85) estimates were calculated when two PLS components included in the model.

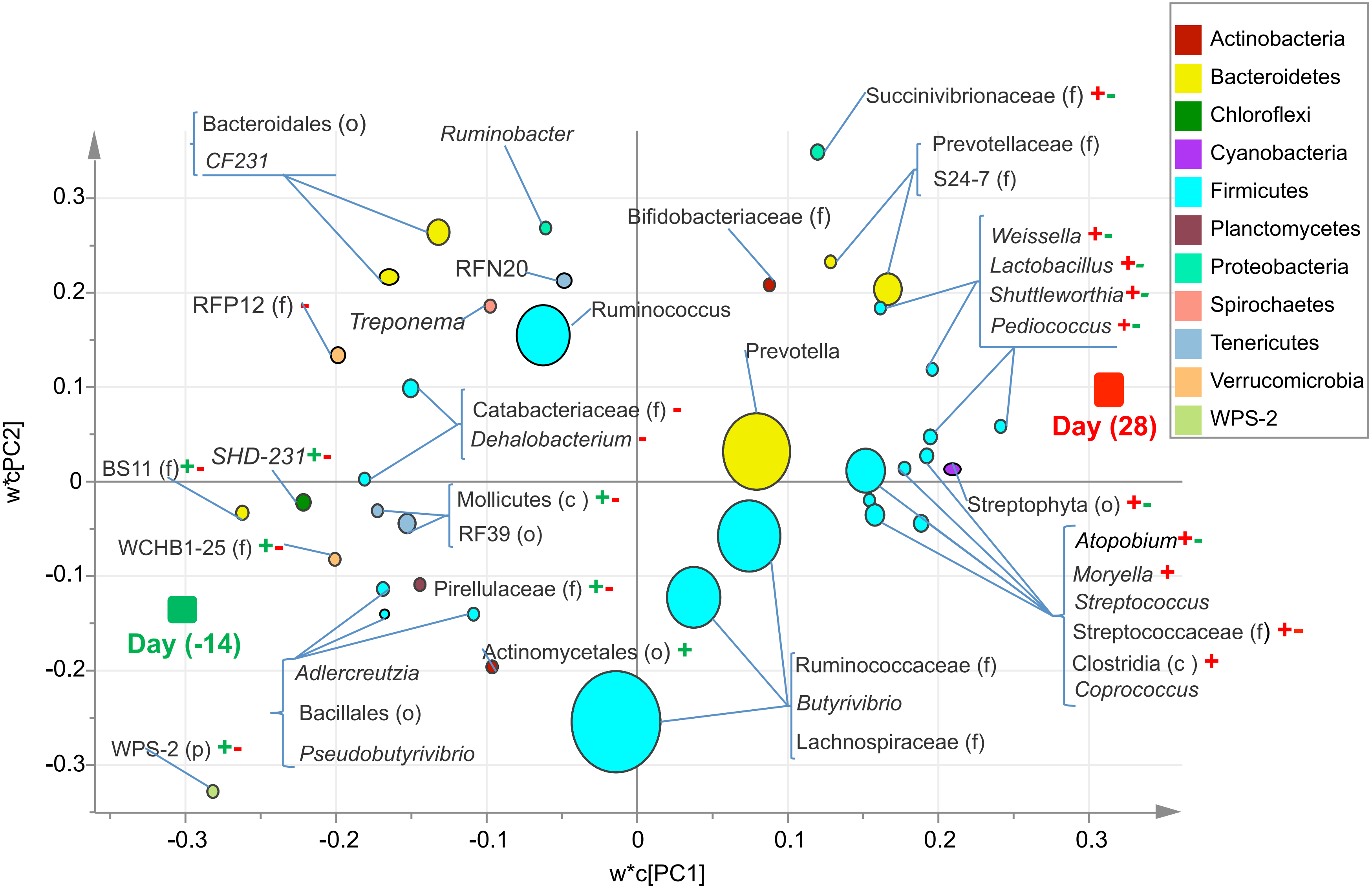

Supplementary figure 2.b. Partial least square discriminant analysis (PLS-DA) of ruminal microbial communities comparing d -14 vs. d 28. Bacterial genera are plotted according to their association with d -14 and d 28, colored based on their corresponding phyla, and sized based on their relative abundances. (+) or (-) indicate genera had positive or negative association with each time point. Some genera could only be affiliated to phylum (P), order (O), or family (F) levels. The R^2 (0.97) and Q^2 (0.76) estimates were calculated when three PLS components included in the model.

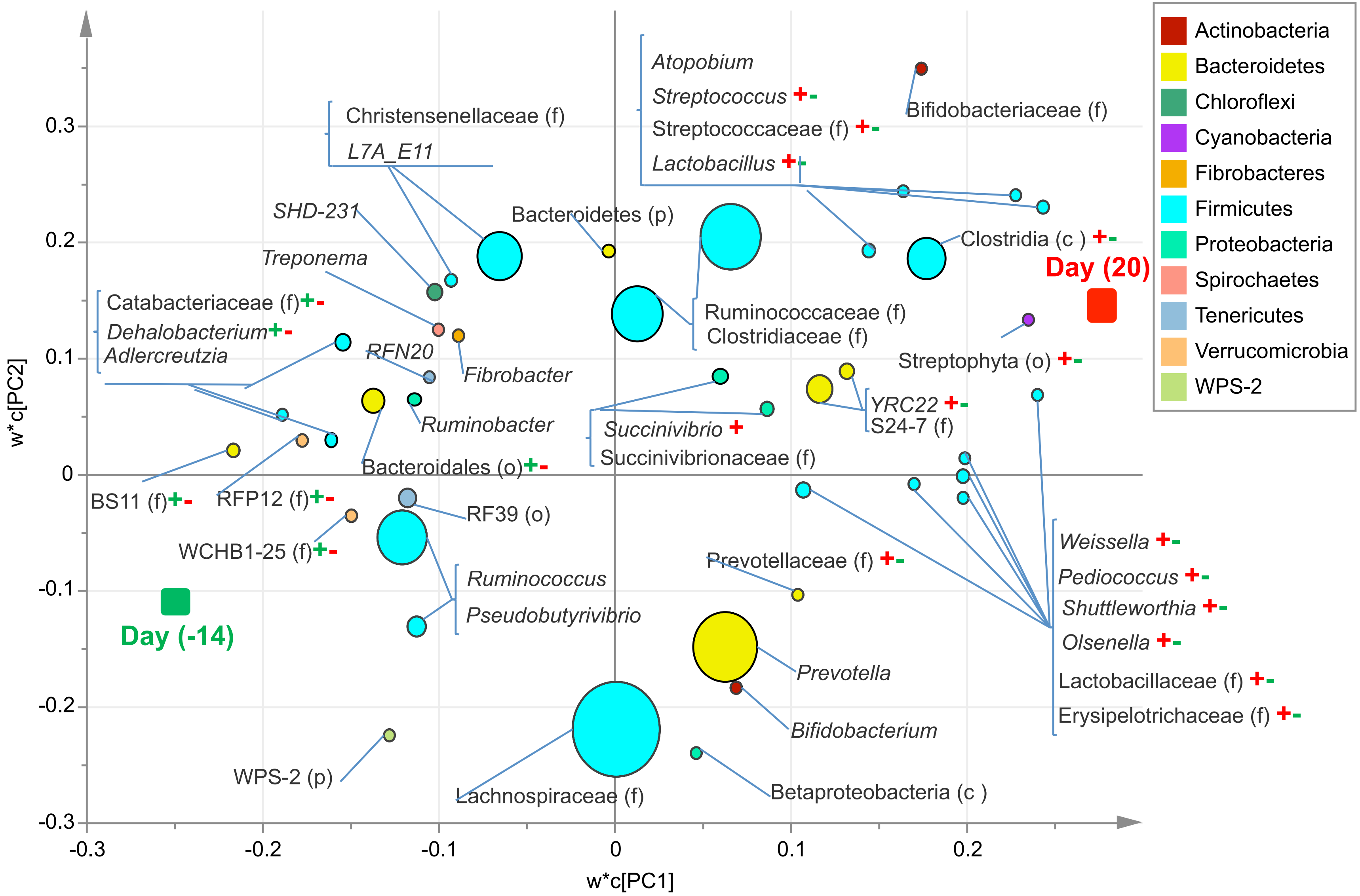

Supplementary figure 2.c. Partial least square discriminant analysis (PLS-DA) of ruminal microbial communities comparing d -14 vs. d 20. Bacterial genera are plotted according to their association with day -14 and day 20, colored based on their corresponding phyla, and sized based on their relative abundances. (+) or (-) signs were used to indicate genera that are significantly associated with each day. Some genera could only be affiliated to phylum (P), order (O), or family (F) levels. The R^2 (=0.95) and Q^2 (=0.74) estimates were calculated when three PLS components included in the model.

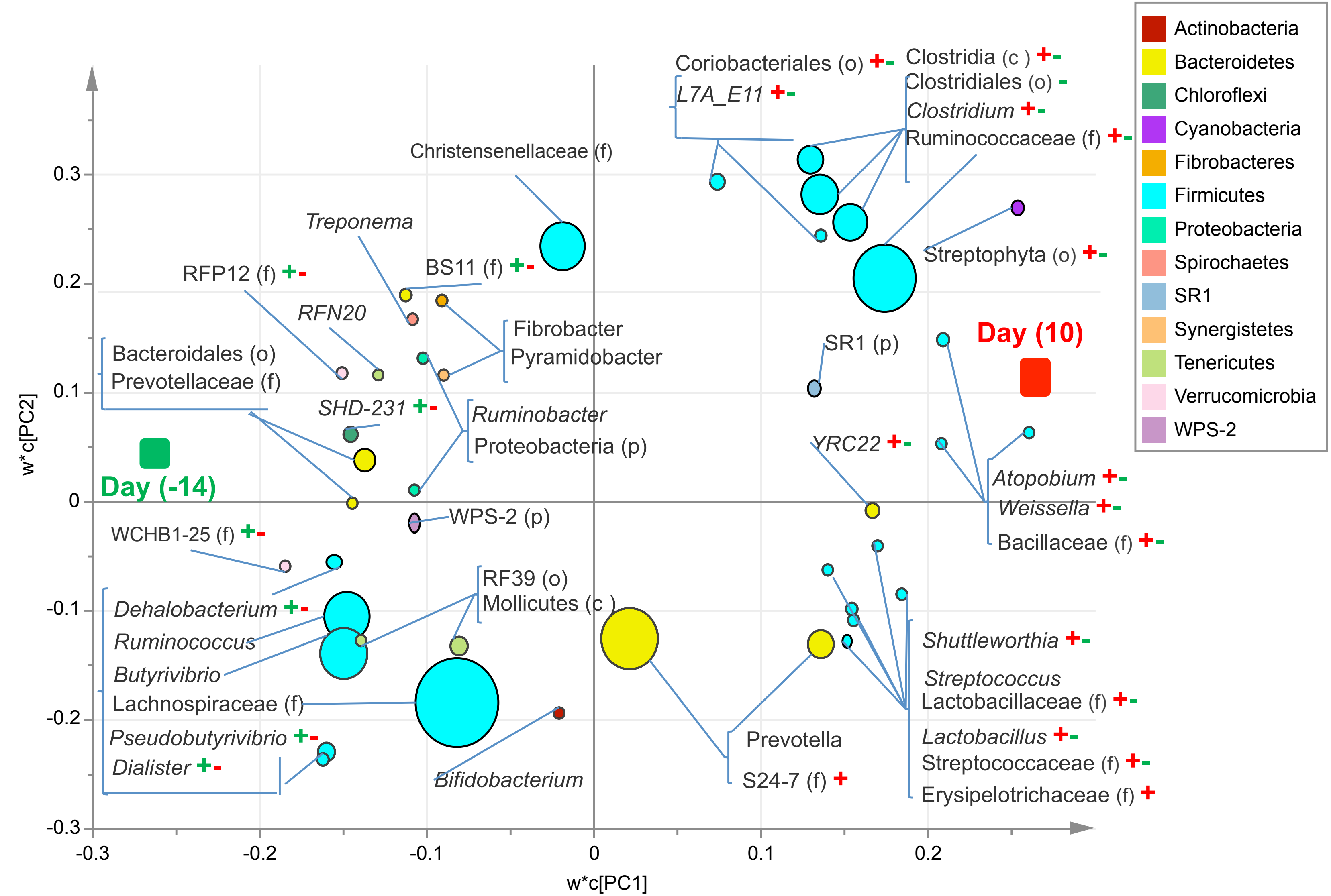

Supplementary figure 2.d. Partial least square discriminant analysis (PLS-DA) of ruminal microbial communities comparing d -14 vs. d 10. Bacterial genera are plotted according to their association with day -14 and day +10, colored based on their corresponding phyla, and sized based on their relative abundances. (+) or (-) signs have been used to indicate genera that are significantly associated with each day. Some genera could only be affiliated to phylum (P), order (O), or family (F) levels. The R^2 (=0.94) and Q^2 (=0.55) estimates were calculated when three PLS components included in the model.

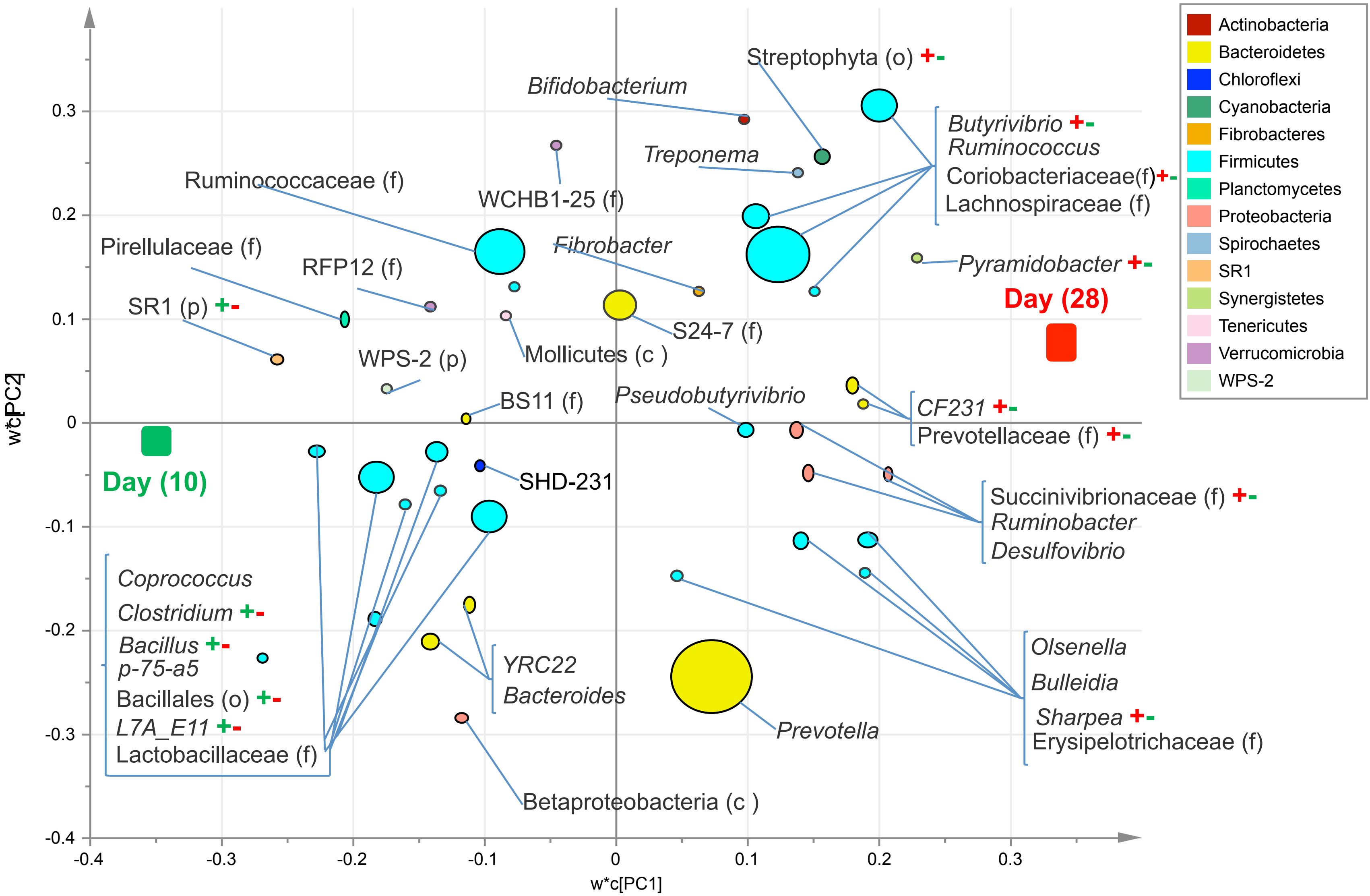

Supplementary figure 2.e. Partial least square discriminant analysis (PLS-DA) of ruminal microbial communities comparing d 10 vs. d 28. Bacterial genera are plotted according to their association with day +10 and day +28, colored based on their corresponding phyla, and sized based on their relative abundances. (+) or (-) signs were used to indicate genera that are significantly associated with each day. Some genera could only be affiliated to phylum (P), order (O), or family (F) levels. The R^2 (=0.96) and Q^2 (=0.59) estimates were calculated when three PLS components included in the model.

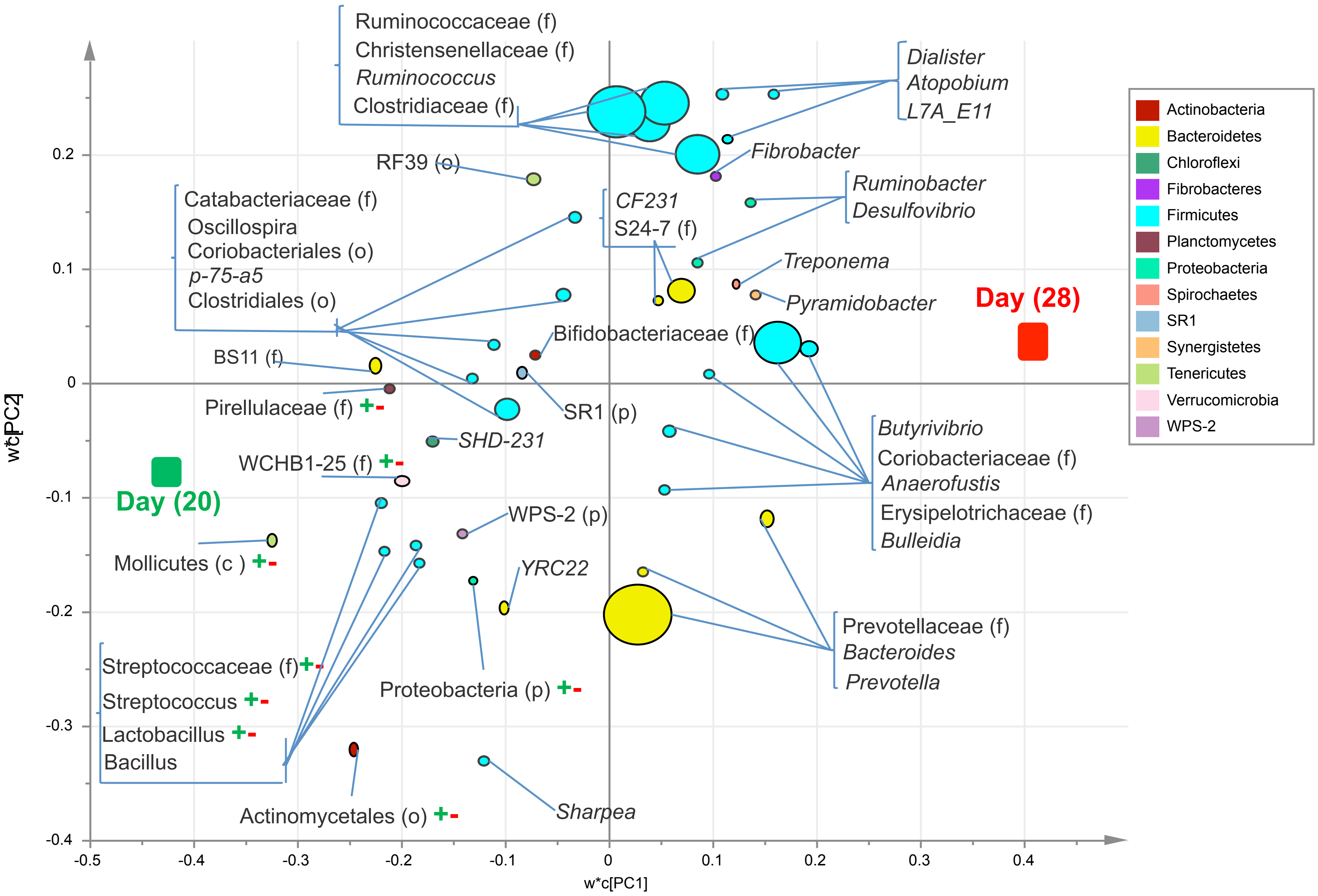

Supplementary figure 2.f. Partial least square discriminant analysis (PLS-DA) of ruminal microbial communities comparing d 20 vs. d 28. Bacterial genera are plotted according to their association with day 20 and day 28, colored based on their corresponding phyla, and sized based on their relative abundances. (+) or (-) signs have been used to indicate genera that are significantly associated with each day and colored based on their corresponding phyla. Some genera could only be affiliated to phylum (P), order (O), or family (F) levels. The R^2 (=0.95) and Q^2 (=0.39) estimates were calculated when three PLS components included in the model.
