# Supplementary material for: Linking Peripartal Dynamics of Ruminal Microbiota to Dietary Changes and Production Parameters
Source: Front Microbiol. 2017 Jan 12;7:2143. doi: 10.3389/fmicb.2016.02143 (PMC5226935; doi:10.3389/fmicb.2016.02143)
Supplement: Supplementary file 5 [file Image3.PDF]

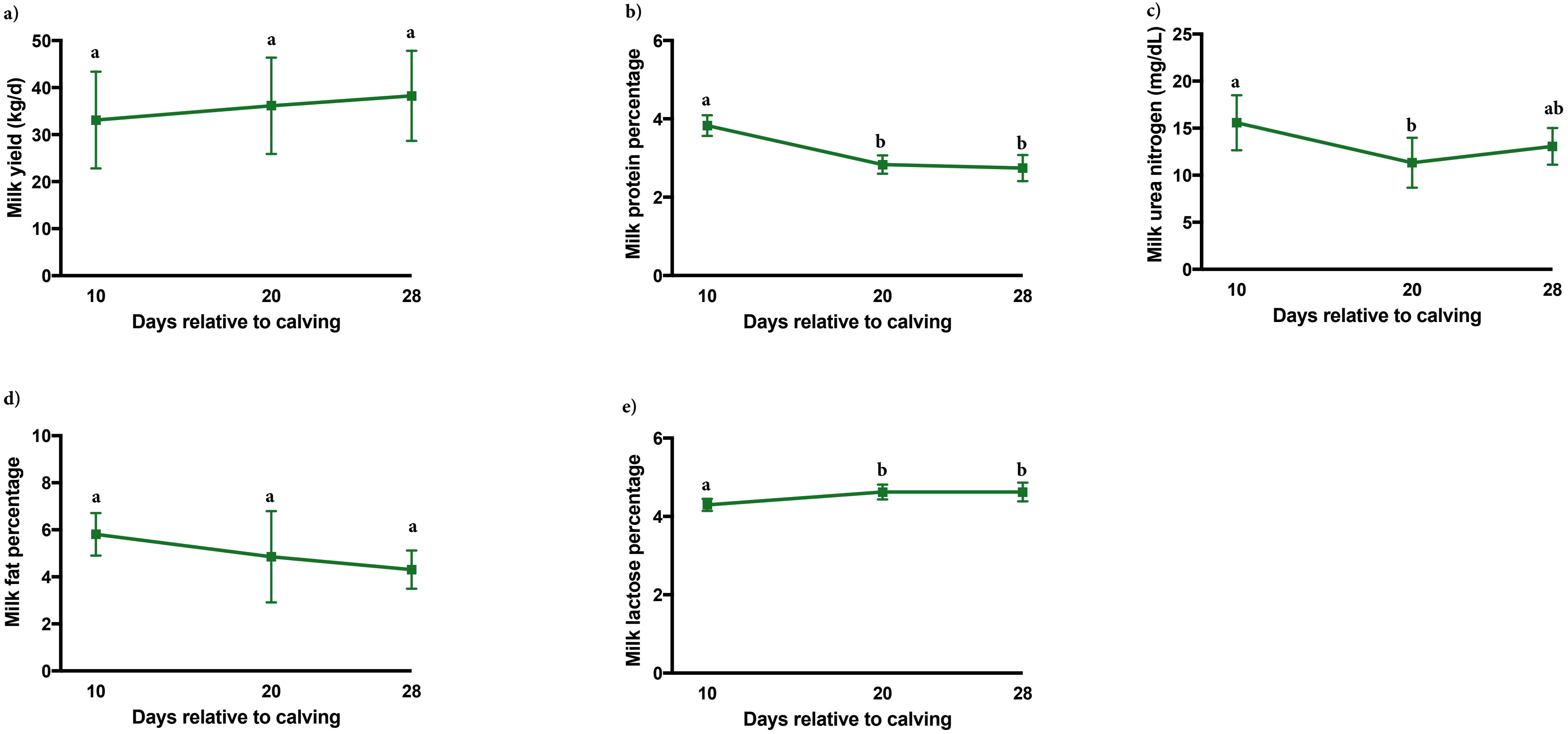

Supplementary figure 3. Postpartal comparisons of the milk yield and milk composition. Figures a-e show the comparison between milk yield, milk protein percentage, milk urea nitrogen content, milk fat percentage, and milk lactose percentage, respectively. Superscripts denote significant differences ($p < 0.05$) between the means. Error bars denote the 95% confidence intervals.
